# Black soldier fly defatted meal as a dietary protein source for broiler chickens: Effects on growth performance, blood traits, gut morphology and histological features

**DOI:** 10.1186/s40104-018-0266-9

**Published:** 2018-07-09

**Authors:** Sihem Dabbou, Francesco Gai, Ilaria Biasato, Maria Teresa Capucchio, Elena Biasibetti, Daniela Dezzutto, Marco Meneguz, Iveta Plachà, Laura Gasco, Achille Schiavone

**Affiliations:** 1 Department of Veterinary Science, University of Turin, Largo Paolo Braccini 2, 10095 Grugliasco, Italy; 2 Institute of Science of Food Production, National Research Council, Largo Paolo Braccini 2, 10095 Grugliasco, Italy; 3 Veterinary Medical Research Institute for Piemonte, Liguria and Valle d’Aosta, Via Bologna 148, 10154 Turin, Italy; 4 Department of Agricultural, Forest and Food Sciences, University of Turin, Largo Paolo Braccini 2, 10095 Grugliasco, Italy; 5 Institute of Animal Physiology, Centre of Bioscience, Slovak Academy of Sciences, Soltesovej 4-6, 040 01 Kosice, Slovak Republic; 6 Institute of Interdisciplinary Research on Sustainability, University of Turin, Via Accademia Albertina 13, 10100 Turin, Italy

**Keywords:** Broiler chickens, Defatted insect meal, Gut morphology, *Hermetia illucens*, Performance

## Abstract

**Background:**

The present study has evaluated the effects of different inclusion levels of a partially defatted black soldier fly (*Hermetia illucens* L.; HI) larva meal on the growth performance, blood parameters and gut morphology of broiler chickens. A total of 256 male broiler chickens (Ross 308) were reared from d 1 to d 35 and assigned to 4 dietary treatments (8 replicates/treatment and 8 birds/replicate). HI larva meal was included at increasing levels (0, 5%, 10% and 15%; HI0, HI5, HI10 and HI15, respectively) in isonitrogenous and isoenergetic diets formulated for 3 feeding phases: starter (1–10 d), growing (10–24 d) and finisher (24–35 d). Two birds per pen were slaughtered at d 35 and morphometric investigations and histopathological alterations were performed.

**Results:**

The live weight (LW) showed linear and quadratic responses to increasing HI larva meal (maximum for HI10 group). Average daily gain (ADG) showed a linear and quadratic responses to HI meal (maximum for HI10 group) during starter and growing periods. A linear decrease was observed for ADG during the finisher period. The daily feed intake (DFI) showed a linear and quadratic effect during the starter period (maximum for HI10 group). Linear and quadratic responses were observed for the feed conversion ratio (FCR) in the growing period and for the whole period of the experiment. The FCR showed a linear response in the finisher period (maximum for HI15). No significant effects were observed for the blood and serum parameters, except for the phosphorus concentration, which showed linear and quadratic responses as well as glutathione peroxidase (GPx) activity, the latter of which showed a linear response. The HI15 birds showed a lower villus height, a higher crypt depth and a lower villus height-to-crypt depth ratio than the other groups.

**Conclusions:**

Increasing levels of dietary HI meal inclusion in male broiler chickens may improve the LW and DFI during the starter period, but may also negatively affect the FCR and gut morphology, thus suggesting that low levels may be more suitable. However, no significant effects on the haematochemical parameters or histological findings were observed in relation to HI meal utilization.

## Background

The growth in population at a global scale has led to a global increase in food consumption patterns as well as changes in life styles and food preferences, which have increased the demand for animal protein [1]. This will affect the demand for livestock feeds, and inevitably place heavy pressure on already limited resources [1]. Therefore, the need for alternative protein sources for livestock is becoming increasingly urgent [2]. Finding alternative sources of poultry feedstuffs is a recent research topic that is still under investigation by nutrition researchers in an attempt to find a balance between high performance and low cost [3]. Makkar et al. [4] mentioned that insects are good candidates to face the global challenge of finding new protein sources at a low cost, especially when taking into account their nutritional value and low breeding space requirements. Scientists have recently started to study insects as innovative feed ingredients for aquaculture [5–8] and poultry [9–16], since the two main derived products (meal and fat) may be suitable for substituting/integrating the conventional protein and fat sources (soybean meal, vegetable oils). The European Commission (EC) has recently approved the use of processed animal proteins obtained from insects in aquafeeds (Regulation 2017/893/EC, 2017), and the Commission is currently working on authorizing their use in poultry feeds. Black soldier fly (*Hermetia illucens* L.; HI) could replace conventional poultry feed ingredients, such as soybean meal, which have a high environmental impact [17]. HI larvae can provide high-value feedstuffs as they are rich in protein (37% to 63%) and have a better amino acid (AA) profile than soybean meal [18]. In addition, they contain a greater amount of lipids (15% to 49%), which can be isolated and used for the preparation of biodiesel, while the rest of the defatted meal could be used as a protein rich source for the feed industry. HI larvae also contain chitin, which is not digestible by monogastric animals [19], and it can negatively affect protein digestibility [20]. Only a few researchers have dealt with the use of defatted HI meals in poultry nutrition, with contrasting results being obtained till now. Schiavone et al. [16] demonstrated that defatted HI meals can be considered as an excellent source of apparent metabolizable energy (AMEn) and digestible AA for broilers, thus potentially resulting into a better efficient nutrient digestion. Marono et al. [21] reported that defatted HI larva meal could also lead to a more favorable feed efficiency in laying hens, even if negative effects can be observed on feed intake and egg production. Differently, Cullere et al. [12] showed that the inclusion of 10% and 15% of defatted HI larva meal in the diet of growing quails (from 10 to 28 d of age) led to comparable productive performances and carcass traits with those of quails fed conventional soybean meal and oil-based diets. Dietary HI meal inclusion has also been reported to positively affect the blood profile of laying hens [21] and Barbary partridges [22], in terms of higher globulin levels [21], lower albumin-to-globulin ratios [21, 22] and lower creatinine levels [22]. Modifications of the dietary protein level [23] and source [24] have been reported to affect the gut histomorphology of broilers to a great extent, to influence their nutrient digestion and absorption, and thus, as a consequence, to condition the growth of the animals [25]. Cutrignelli et al. [26] have recently investigated the intestinal morphometry of laying hens fed HI larva meal, finding significant differences between the animals fed with insect meal and those fed with the standard diet. The authors showed higher villi height in the duodenum of HI groups than the standard diet.

However, despite the growing research interest about the modulation of animal performance, and gut histomorphology by dietary HI meal utilization in avian species, such information is still lacking in broilers.

Therefore, on the basis of the above reported background, this research has evaluated the effects of a defatted HI larva meal on the growth performance, blood parameters and gut morphology of broiler chickens, in order to (1) evaluate the influence of insect meal utilization on animal performance, to (2) investigate the impact of insect meal utilization on intestinal morphology and its relationship with the performance, and to (3) determine the optimal inclusion level of HI larva meal for broiler chickens.

## Methods

### Birds and husbandry

The current study has been performed in the poultry facility of the Department of Agricultural, Forest and Food Sciences (DISAFA) of the University of Turin (Italy). The poultry house (wide × long × high) was 7 m × 50 m × 7 m, equipped with a waterproof floor and walls, and was covered completely by tiles and with an automatic ventilation system. A total of 256 male broiler chickens (Ross 308) were reared from d 1 to d 35 and assigned to 4 dietary treatments (8 pens/treatment and 8 birds/pen). Each pen (wide × long) was 1.0 m × 1.5 m and was equipped with a feeder, an automatic drinker and rice hulls as bedding. During the first 3 wk, the animals were heated by infrared lamps to maintain the temperature recommended for standard breeding practices (Aviagen: Ross broiler management handbook) [27]. The lighting schedule was 18 h light: 6 h dark for the whole experimental period. At hatching, the chicks were vaccinated against Newcastle disease, Marek disease, infectious bronchitis and coccidiosis.

### Insect meal and diets

The study was carried out to evaluate the effect of the dietary inclusion of partially defatted HI larva meal in broiler chickens diets. The diets were obtained by including, as a feed basis, increasing levels of HI larva meal (0, 5%, 10% and 15%; HI0, HI5, HI10 and HI15, respectively). A partially defatted HI larva meal, obtained by processing larvae reared on a vegetable by-product substrate, was purchased from Hermetia Deutschland GmbH & Co. KG, Baruth/Mark (Germany). The diets were split into three phases for each treatment: a starter diet (1–10 d), a grower diet (10–24 d) and a finisher diet (24–35 d). The diets met or exceeded the NRC [28] requirements and were adjusted according to the Aviagen [27] broiler nutrition specifications. The experimental diets were isonitrogenous and isoenergetic for each phase. The diets were formulated on the basis of the chemical and AA compositions, and of the AMEn value for HI larva meal, as outlined in Schiavone et al. [16], while for the other ingredients according to INRA [29].

Feeds and water were provided ad libitum throughout the trial. Any clinical signs of illness or mortality were monitored daily throughout the experimental period. The ingredients of the experimental diet are reported in Table 1.

**Table 1:** Ingredients, apparent metabolizable energy and chemical composition of the experimental diets

Items	Starter period				Growing period				Finisher period			
	HI0	HI5	HI10	HI15	HI0	HI5	HI10	HI15	HI0	HI5	HI10	HI15
Ingredients, g/kg as fed												
Maize meal	508.8	526.9	545.5	566.9	536.1	557.1	574.5	606.1	537.2	590.8	620.3	653.2
Soybean meal (48% CP)	345.3	299	248	193	332	277.4	230	153	300	253	178	100
HI larvae meal	0	50	100	150	0	50	100	150	0	50	100	150
Corn gluten meal	54	32	15	0	35	20	0	0	21	0	0	0
Soybean oil	46.3	45.1	43	40	59.3	56.4	54.9	48	71.2	70	63.2	56
Dicalcium phosphate	6.5	8.5	10.5	12.5	4	6	8.3	10.6	3.5	6	8.3	10.5
Calcium carbonate	17.3	16.4	15.6	14.8	16.7	15.8	14.8	14	15.1	14	13.2	12.4
Sodium chloride	2.3	2.3	2.3	2.3	2.3	2.3	2.3	2.3	2.3	2.3	2.3	2.3
Sodium bicarbonate	1.3	1.3	1.3	1.3	1.3	1.3	1.3	1.3	1.3	1.3	1.3	1.3
*DL*-Methionine	2	2.2	2.3	2.3	1.8	1.9	2	2	1.8	1.9	1.9	1.8
*L*-Lysine	5.1	5.2	5.4	5.8	3.8	4.1	4.2	5	3.3	3.4	4.1	5
Threonine	1.9	1.9	1.9	1.9	1.5	1.5	2	2	1.1	1.1	1.2	1.3
Trace mineral-vitamin premix^a^	8	8	8	8	5	5	5	5	5	5	5	5
Choline chloride	0.2	0.2	0.2	0.2	0.2	0.2	0.2	0.2	0.2	0.2	0.2	0.2
3-Phytase	1	1	1	1	1	1	1	1	1	1	1	1
Total	100	100	100	100	100	100	100	100	100	100	100	100
AMEn^b^, MJ/kg DM	12.56	12.56	12.56	12.56	12.98	12.98	12.98	12.98	13.4	13.4	13.4	13.4
Nutrient composition^c^, %												
CP	23.01	22.98	23.01	23.01	21.50	21.52	21.52	21.52	19.51	19.47	19.54	19.50
EE	7.30	7.33	7.28	7.14	8.63	8.50	8.50	8.01	9.86	9.89	9.41	8.88
CF	3.25	2.99	2.70	2.41	3.21	2.91	2.64	2.25	3.08	2.82	2.43	2.04
Calcium	0.96	0.96	0.96	0.96	0.87	0.87	0.87	0.87	0.79	0.79	0.79	0.79
Phosphorus	0.48	0.48	0.48	0.48	0.43	0.43	0.43	0.43	0.40	0.40	0.40	0.40

*HI* Hermetia illucens, *AMEn* apparent metabolizable energy, *CP* crude protein, *EE* ether extract, *CF* crude fiber, *DM* dry matter

^a^Mineral-vitamin premix*:* vitamin A (retinyl acetate), 12,500 IU; vitamin D_3_ (cholecalciferol), 3000 IU; vitamin E (*DL*-a-tocopheryl acetate), 60 IU; vitamin K (menadione sodium bisulfite), 1.02 mg; riboflavin, 2.0 mg; pantothenate, 8.0 mg; niacin, 6 mg; piridossin, 4 mg; folic acid, 0.5 mg; biotin, 0.10 mg; tiamin, 1.0 mg; vitamin B_12_, 20 mg; Mn, 120 mg; Zn, 80 mg; Fe, 52 mg; Cu, 15 mg; I, 1.5 mg; Se, 0.4 mg

^b^Calculated according to Schiavone et al. [16] and INRA [29]

^c^Chemical analyses were carried out on three replicates of each feed sample

### Chemical composition of the feeds

The proximate composition of the experimental diets is shown in Table 1. Feed samples were ground using a cutting mill (MLI 204; Bühler AG, Uzwil, Switzerland) and analyzed to establish the crude protein (CP; AOAC #984.13), crude fiber (CF; AOAC #978.10), calcium (AOAC #927.02) and phosphorous (AOAC #965.17) contents according to AOAC International [30]. Ether extract (EE; AOAC #2003.05) was analyzed according to AOAC International [31].

### Growth performance

Health status and mortality were monitored daily throughout the whole experimental period. The live weight (LW) of the animals was recorded at an individual level at the beginning of the trial, and on d 10, 24 and 35. The average daily gain (ADG) and average daily feed intake (DFI) were recorded at an individual and at a pen level, respectively, at the end of each growth period. The feed conversion ratio (FCR) was determined for each growth period and for the overall experimental period. All the measurements were made on a pen basis using high precision electronic scales (Sartorius – Signum®).

### Haematological and serum parameters

At 35 d of age, on the basis of the average final body weight, 2 birds per pen were slaughtered from each feeding group. Blood samples were collected from the identified broilers: 2.5 mL was placed in an EDTA tube and 2.5 mL in a serum-separating tube. A blood smear was prepared, using one glass slide for each bird, from a drop of blood without anticoagulant. The smears were stained using May-Grünwald and Giemsa stains [32]. The total red and white blood cell counts were determined in an improved Neubauer haemocytometer on blood samples previously treated with a 1:200 Natt-Herrick solution. One hundred leukocytes, including granular (heterophils, eosinophils and basophils) and non-granular (lymphocytes and monocytes) leukocytes were counted on the slide, and the heterophils-to- lymphocytes (H/L) ratio was calculated. The tubes without anticoagulant were left to clot, in a standing position, at room temperature for approximately 2 h to obtain serum. The serum was separated by means of centrifugation of 700×*g* for 15 min and frozen at − 80 °C until analysis. The electrophoretic pattern of the serum was obtained using a semi-automated agarose gel electrophoresis system (Sebia Hydrasys®, Norcross, GA, the USA). The urea, uric acid, aspartate aminotransferase (AST), creatinine, triglyceride, cholesterol, calcium, phosphorus, magnesium and iron serum concentrations were measured by means of enzymatic methods in a clinical chemistry analyzer (Screen Master Touch, Hospitex diagnostics Srl., Florence, Italy). The total antioxidant status (TAS) in the plasma, the haemoglobin (Hb) content and the glutathione peroxidase (GPx, EC 1.11.1.9) activity in the blood were analyzed using commercial kits (Randox, the UK).

### Morphological investigations

The slaughtered animals were submitted to anatomopathological investigations. Intestinal segment samples (approximately 5 cm in length) of the duodenum, jejunum and ileum were excised and flushed with 0.9% saline to remove the entire content. The collected segments of intestine were the loop of the duodenum, the tract before Meckel’s diverticulum (jejunum) and the tract before the ileocolic junction (ileum). Samples of liver, spleen, thymus, bursa of Fabricius, kidneys and heart were also collected. Gut segments were fixed in Carnoy’s solution for morphometric analysis, while the other organ samples were fixed in a 10% buffered formalin solution for histopathological examination. Tissues were routinely embedded in paraffin wax blocks, sectioned at 5 μm thickness, mounted onto glass slides and stained with Haematoxylin & Eosin (HE). The evaluated morphometric indices were the villus height (Vh, from the tip of the villus to the crypt), crypt depth (Cd, from the base of the villus to the submucosa) and the villus height-to-crypt depth (Vh/Cd) ratio [23]. Morphometric analyses were performed on 10 well-oriented and intact villi and 10 crypts chosen from the duodenum, jejunum and ileum [24]. The following histopathological alterations were evaluated: white pulp hyperplasia and depletion in the spleen, cortical depletion in the thymus, follicular depletion and intrafollicular cysts in the bursa of the Fabricius and lymphoid tissue activation in the liver [11]. The heart and kidneys were assessed for inflammatory and degenerative diseases. The observed histopathological findings were evaluated using a semiquantitative scoring system, as previously assessed by Biasato et al. [11]: absent/minimal (score = 0), mild (score = 1) and severe (score = 2).

### Statistical analysis

The statistical analyses were performed using the SPSS software package (version 21 for Windows, SPSS Inc., Chicago, IL, USA). Shapiro-Wilk’s test established normality or non-normality of distribution. The experimental unit was a pen for growth performance, while the individual bird was used for the blood parameters, morphometric data and histopathological findings. The collected data were tested by means of one-way ANOVA. Polynomial contrasts were used to test the linear and quadratic responses to increases in the HI inclusion level in the diet. Intestinal morphometric indices were analyzed by fitting a general linear model (GLM). The GLM allowed the morphometric indices (Vh, Cd and Vh/Cd, separately) to depend on three fixed factors (diet, intestinal segment and interaction between diet and intestinal segment). The interactions between the levels of the fixed factors were evaluated by means of pairwise comparisons. Statistical analysis was performed according to the “General Linear Models > Univariate” procedure. Histopathological scores were analyzed by means of the Kruskal-Wallis test (post-hoc test: Dunn’s Multiple Comparison test). Significance was declared at *P* <  0.05. A statistical trend was considered for *P* <  0.10. The results are presented as the mean and standard error of the means (SEM).

## Results

### Growth performance

During the whole experimental period, the birds showed all their vitalities (no signs of illness were observed, and the mortality rate was zero in all the groups). Table 2 summarizes the growth performance of the broiler chickens. The initial LW of the chicks did not differ (*P* > 0.05) for the different dietary treatments. At 10, 24 and 35 d of age, the LW showed a linear and quadratic responses to HI meal with a maximum observed for HI10 group (*P* <  0.05). Similar results were observed for ADG during the study. ADG showed a linear (*P* = 0.005 and *P* <  0.001) and quadratic (*P* = 0.031 and *P* <  0.001) responses to HI meal with a maximum observed for HI10 group from 1 to 10 and from 10 to 24 d of age. On the other hand, a linear decrease (*P* = 0.007) was elicited for the HI meal as the level of supplementation increased from 0 to 15% during the finisher period. DFI increased linearly (*P* = 0.012) and quadratically (*P* = 0.040) from 1 to 10 d of age, with a maximum observed for HI10 group. DFI instead showed no differences (*P* > 0.05) in the periods from 10 to 24 d and 24 to 35 d of age. In the period from 1 to 10 d of age, FCR was similar for the dietary treatments (*P* > 0.05). Linear and quadratic responses (*P* <  0.001) were observed for FCR between the groups in the periods from 10 to 24 d of age and from 1 to 35 d of age, with a maximum corresponding to the inclusion of 15% of HI meal. FCR instead showed a linear response to increasing HI meal levels in the period from 24 to 35 d of age (*P* = 0.011), with a maximum corresponding to the inclusion of 15% of HI meal.

**Table 2:** Effect of the dietary HI larva meal inclusion level on the growth performances of the broiler chickens (*n* = 8)

Items	Age	Dietary treatments				SEM	*P*-value	
		HI0	HI5	HI10	HI15		Linear	Quadratic
LW, g	1 d	40.23	40.20	40.33	40.23	0.020	0.443	0.363
	10 d	262.19	259.22	285.23	267.89	2.349	0.005	0.030
	24 d	1194.91	1199.45	1227.89	1095.23	10.157	< 0.001	< 0.001
	35 d	2269.25	2264.11	2278.95	2072.15	18.173	< 0.001	< 0.001
ADG, g	1–10 d	22.20	21.90	24.49	22.77	0.234	0.005	0.031
	10–24 d	66.62	67.16	67.33	59.10	0.716	< 0.001	< 0.001
	24-35d	97.67	96.79	95.55	88.81	1.187	0.007	0.179
DFI, g	1–10 d	25.89	25.64	28.52	26.54	0.279	0.012	0.040
	10–24 d	97.50	96.69	98.70	95.23	0.572	0.340	0.238
	24–35 d	176.42	174.62	174.27	171.73	0.988	0.115	0.852
FCR, g/g	1–10 d	1.17	1.17	1.16	1.17	0.005	0.866	0.900
	10–24 d	1.46	1.44	1.47	1.61	0.016	< 0.001	0.001
	24–35 d	1.81	1.81	1.83	1.93	0.018	0.011	0.136
	1–35 d	1.60	1.59	1.60	1.72	0.012	< 0.001	< 0.001

*HI Hermetia illucens*, *SEM* standard error of the mean, *LW* live weight, *ADG* Average daily gain, *DFI* daily feed intake, *FCR* feed convertion ratio

### Blood parameters

The haematological and serum biochemical parameters of the broiler chickens are summarized in Table 3. No significant effects related to HI meal utilization were observed for the blood or serum parameters (*P* > 0.05), except for the phosphorus concentration, which showed a linear and quadratic responses to increasing HI meal levels (*P* < 0.001), with a maximum corresponding to the HI10 group, and for the activity of GPx in the blood, which showed a linear response (*P* = 0.002) to increasing HI meal levels for up to 15%. A linear response trend to increasing HI meal levels was also determined for up to 15% of triglycerides and TAS (*P* = 0.057 and *P* = 0.054, respectively).

**Table 3:** Effect of the dietary HI larva meal inclusion level on the haematological and serum parameters of the broiler chickens (*n* = 16)

Items	Dietary treatments				SEM	*P*-value	
	HI0	HI5	HI10	HI15		Linear	Quadratic
Erythrocyte, 10^6^ cell/μL	4.18	3.94	3.93	3.98	0.046	0.132	0.122
Leukocyte, 10^3^ cell/μL	13.70	13.55	13.77	14.32	0.246	0.344	0.492
H/L ratio	0.66	0.68	0.65	0.69	0.028	0.838	0.824
Urea, g/dL	34.88	39.53	42.59	36.30	1.980	0.712	0.177
Uric Acid, mg/dL	2.89	2.67	2.46	2.77	0.180	0.750	0.477
AST, IU/L	379.57	367.52	366.33	358.78	13.508	0.437	0.904
Creatinine, mg/dL	0.29	0.27	0.27	0.28	0.006	0.546	0.542
Triglycerides, mg/dL	105.25	94.70	97.82	84.32	3.541	0.057	0.826
Cholesterol, mg/dL	81.34	81.07	85.73	82.64	2.044	0.660	0.734
Calcium, mg/dL	8.77	7.56	8.43	8.42	0.209	0.947	0.155
Phosphorus, mg/dL	3.91	5.90	6.02	5.34	0.172	< 0.001	< 0.001
Magnesium, mEq/L	1.12	1.04	0.96	0.92	0.055	0.186	0.837
Iron, μg/dL	59.89	61.12	66.03	61.72	3.866	0.780	0.727
TAS, mmol/L	0.58	0.65	0.79	0.78	0.044	0.054	0.679
GPx, U/g Hb	88.68	107.27	155.52	178.14	11.978	0.002	0.884

*HI Hermetia illucens*, *SEM* standard error of the mean, *H/L*, heterophiles to lymphocytes ratio, *AST* aspartate amino transferase, *TAS* Total antioxidant status, *GPx* glutathione peroxidase, *Hb* haemoglobin

### Morphological investigations

The effects of the diet, intestinal segment and interaction between the diet and intestinal segment on the intestinal morphometric indices are summarized in Tables 4 and 5. Vh and Cd depended on the diet (*P* < 0.05) and intestinal segment (*P* < 0.001), while no influence of interaction between the diet and intestinal segment (*P* > 0.05) was observed on the morphometric index (Table 4). The HI15 birds showed lower Vh than the other groups (*P* < 0.05) and higher Cd than HI0 and HI5 (*P* < 0.05). Furthermore, higher Vh and Cd were found in the duodenum than in the other gut segments (*P* < 0.001 and *P* < 0.01, respectively) (Table 5). The diet (*P* < 0.01), intestinal segment (*P* < 0.001) and the interaction between the diet and intestinal segment (*P* < 0.05) all influenced (*P* < 0.05) the Vh/Cd (Table 4). The HI15 birds in particular showed lower Vh/Cd than the other groups (*P* < 0.01). Furthermore, a higher Vh/Cd was found in the duodenum than in the other gut segments (*P* < 0.01) (Table 5).

**Table 4:** Effects of diet, intestinal segment and interaction between diet and intestinal segment on the intestinal morphometric indices of the broiler chickens (*n* = 16)

Index	Fixed effect	d.f.^c^	F	*P*-value
Vh, mm	Diet^a^	3	2.893	0.038
	Intestinal segment^b^	2	53.448	< 0.001
	Diet × Intestinal segment	6	0.922	0.482
Cd, mm	Diet	3	3.223	0.040
	Intestinal segment	2	10.130	< 0.001
	Diet × Intestinal segment	6	1.988	0.072
Vh/Cd, mm/mm	Diet	3	5.024	0.002
	Intestinal segment	2	17.858	< 0.001
	Diet × Intestinal segment	6	2.274	0.040

*Vh* villus height, *Cd* crypt depth, *Vh/Cd* villus height to crypt depth ratio

^a^Four dietary treatments: HI0 = 0% inclusion level of *Hermetia illucens*; HI5 = 5% inclusion level of *Hermetia illucens*; HI10 = 10% inclusion level of *Hermetia illucens*; HI15 = 15% inclusion level of *Hermetia illucens*

^b^Three intestinal segments: duodenum, jejunum and ileum

^c^Degrees of freedom

**Table 5:** Least square means of the intestinal morphometric indices of the broiler chickens in relation to the diet and intestinal segment (*n* = 16)

Index	Fixed effect	Effect levels	Least square mean^a^	SEM
Vh, mm	Diet	HI0	1.66^a^	0.05
		HI5	1.64^a^	
		HI10	1.70^a^	
		HI15	1.49^b^	
	Intestinal segment	DU	1.99^a^	0.05
		JE	1.57^b^	
		IL	1.30^c^	
Cd, mm	Diet	HI0	0.16^a^	0.01
		HI5	0.17^a^	
		HI10	0.17^ab^	
		HI15	0.19^b^	
	Intestinal segment	DU	0.18^a^	0.00
		JE	0.16^ab^	
		IL	0.15^b^	
Vh/Cd, mm/mm	Diet	HI0	10.79^a^	0.41
		HI5	10.83^a^	
		HI10	10.45^a^	
		HI15	8.89^b^	
	Intestinal segment	DU	11.71^a^	0.36
		JE	10.14^b^	
		IL	8.79^c^	

*HI Hermetia illucens*, *Vh* villus height, *Cd* crypt depth, *Vh/Cd* villus height to crypt depth ratio, *DU* duodenum, *JE* jejunum, *IL* ileum

^a^Means with different superscript letters (a, b) within the same column per fixed effect (i.e. diet, intestinal segment) differ significantly (*P* < 0.05)

Regardless of the dietary treatment, histopathological alterations developed in the spleen, thymus, bursa of Fabricius and liver. The heart and kidneys instead showed no significant alterations. The histopathological scores of the broiler chickens are shown in Table 6. The inclusion of dietary HI larva meal did not significantly affect (*P* > 0.05) the grading of the histopathological alterations.

**Table 6:** Effects of dietary *Hermetia illucens* (HI) larva meal inclusion level on the histopathological scores of the broiler chickens (*n* = 16)

	HI0	HI5	HI10	HI15	SEM	*P*-value
Spleen	1.08	1.17	1.33	0.83	0.09	0.259
Thymus	0.17	0.00	0.25	0.33	0.06	0.312
Bursa of Fabricius	1.08	0.75	0.75	0.75	0.09	0.430
Liver	0.92	0.50	0.92	0.33	0.11	0.174
Heart	No alterations					
Kidney	No alterations					

Data are expressed as mean values of the scores attributed to the histopathological alterations (0 = absent/minimal; 1 = mild; 2 = severe)

*HI Hermetia illucens*, *SEM* standard error of the mean

## Discussion

To the best of the authors’ knowledge, the current study is the first to have tested partially defatted HI larva meal in the diets of broiler chickens.

### Growth performance

Dietary HI inclusion has positively influenced the growth performance of the birds in the present trial up to 10%, in terms of improved LW and DFI during the starter period. The increased DFI observed in the starter period, which was accompanied by increased LW and ADG and unaffected FCR, was quite relevant. The starter period is considered the most important in broiler production, since growth and development take place at an incredible rate in this phase. In fact, the chicks’ weight quadruples, thus influencing the subsequent growth rate [27, 33]. It appears that the inclusion of 10% larva meal in partial substitution of soybean meal is suitable, as a feed ingredient, for broiler chicken diets during the starter period. The positive modulation of LW and DFI by dietary HI meal inclusion partially agrees with what reported by Oluokon [34] and Loponte et al. [22], who observed improved growth rate and higher LW in chickens and Barbary partridges, respectively, fed with HI meal as a component of a complete diet and as partial replacement (25% or 50%) of soybean meal. This is also in agreement with the successful use of HI larvae and prepupae grown on swine manure or kitchen waste as feed additives in young chicks [35]. The increased DFI and LW observed in the birds of present study can be attributed to the improvement of the diet palatability related to HI meal inclusion level. Cullere et al. [12] previously performed a feed-choice test in broiler quails and observed that the birds tended to prefer the diets including HI meal, thus potentially confirming the chickens innate behavior of consuming insects when reared in free-range systems [36]. Despite these positive effects, the FCR of the growing and finisher periods was impaired in the animals fed HI meal of the present study, but only at the 15% supplemental level. This appears to be in contrast with the previous studies currently available. Indeed, Marono et al. [21] and Loponte et al. [22] observed a better feed efficiency in laying hens and Barbary partridges, respectively, fed with HI as complete replacement of soybean meal and fed with *Tenebrio molitor* (TM) as partial replacement (25% or 50%) of soybean meal. Unaffected FCR has also been reported in broiler chickens fed diets with increasing levels of defatted HI meal [37], in laying hens fed with partially defatted HI larva meal as partial or complete replacement of soybean cake [38] and in growing broiler quails fed with 10% and 15% inclusion levels of defatted HI larva meal as partial replacement of soybean meal and oil [12]. Al Qazzaz et al. [39] also reported unaffected or improved growth performance and productivity in laying hens fed diets supplemented with 1% or 5% of HI larva meal. The impaired feed efficiency observed in the broiler chickens fed 15% HI diet of the current trial may be attributed to the chitin contained in HI larvae, which is not digestible by monogastric animals [19], and it can negatively affect protein digestibility [20].

The results obtained in the present study can be contextualized within the wide variability of the findings reported in the above mentioned studies. This heterogeneity may be related to the nutritive value of the insect meal that was used, which can be influenced by the insect life stage (adult, larva or pupa), the insect rearing substrate, the defatting process [19] and also the growing period in which the chickens were fed.

### Haematological and serum parameters

All of the blood parameters obtained in the present trial suggested that a partially defatted HI larva meal does not affect the health status of animals. Furthermore, all the blood parameters obtained in the present study fell within the physiological ranges [40]. The H/L ratio has been used for the measurement of distress conditions in chickens, where an increased H/L ratio may indicate that the animals suffered from infections, inflammation or stress [41–43]. The H/L ratio of chickens fed HI larva meal did not show any significant differences from the HI0 group. No significant differences (*P* > 0.05) were found for creatinine, thus implying that HI larva meal has similar effects on the kidney functions of the birds. The higher phosphorus serum concentrations observed in the birds fed HI diets could be related to the higher bioavailability of phosphorus in HI larva meal than in plant ingredients [44]. The phosphorus content of HI larvae has been reported to be closely correlated with the phosphorus content of the provided substrate [17, 45]. Differently from what observed in the present study, dietary HI meal inclusion has been reported to positively affect the blood profile of laying hens [21] and Barbary partridges [22]. However, some studies concerning other insect meal species, such as TM [9, 11, 13, 14], did not show any significant difference in the blood traits of the animals, thus confirming our findings.

The main function of GPx, as part of the antioxidative defense system, is the removal or detoxification of hydrogen peroxides, and this enzyme serves as a major defence mechanism against oxidative damage. In this way, the enhancement of GPx activity can inhibit the production of reactive species. Many different dietary factors can improve GPx activity, although selenium supplementation exerts the main positive effects [46]. However, GPx activity may also be enhanced by other dietary antioxidant molecules such as vitamin E [47]. Insect meal can provide dietary tocopherols (42.72 mg/kg of insect meal) as reported by Secci et al. [48]. In light of these considerations, it is possible to hypothesize that the increasing GPx activity in HI groups found in our study could have been related to the dietary antioxidant contribution of the insect meal.

### Morphological investigations

Dietary HI meal inclusion has been found to partially affect the intestinal morphology of the broiler chickens in the current study. In particular, shorter villi, deeper crypts and reduced Vh/Cd were observed in the HI15 group than in the other groups. This is in agreement with the previous findings of Biasato et al. [14], who observed lower Vh, higher Cd and decreased Vh/Cd in broiler chickens fed a 15% level of TM meal inclusion than a control and 5% TM diets. Similar findings were also reported by Cutrignelli et al. [26] in jejunum and ileum of laying hens fed diets with HI meal as complete replacement of soybean meal. It is well known that the morphology of the small intestine, especially regarding the crypts and villi of the absorptive epithelium, plays a key role in the final phase of nutrient digestion and assimilation [25]. The simultaneous observation of short villi and deep crypts is indicative of negative gut development. On one hand, shorter villi present a reduced surface area for the absorption of nutrients [49], and are indicators of a decreased villus function [50]. On the other hand, larger crypts may be related to an increase in cell turn-over, which also involves an increase in energy requirement for gut maintenance, with subsequent utilization of the nutrients for the functioning of the digestive tract instead of for growth [51]. Qaisrani et al. [24] observed that lower villus heights and greater crypt depths may lead to poor digestion and less absorption of nutrients and, as a consequence, a poor performance of the animals, and they attributed this to the modifications of the protein source. Undigested protein may in fact increase the quantity of undigested AAs, with a subsequent enhanced proteolytic fermentation by the resident microbiota [52] and formation of toxic compounds, such as amines, ammonia, skatole or indoles [53]. It is well known that the chitin contained in the exoskeleton of insects can negatively influence the nutrient digestibility of CP [54, 55]. A moderate CP digestibility (62%) has also been reported for defatted HI larva meal [16]. Therefore, the altered morphometric indices observed in male broilers fed a 15% level of HI meal inclusion could be related to the higher level of insects in this diet, thus also suggesting that lower levels may be preferable. This hypothesis seems to be supported by the results of Biasato et al. [11, 14], who found no modifications of the gut morphometric indices in free-range or broiler chickens fed diets including 7.5% and 5% inclusions of TM meal, respectively. A similar consideration can also be made in relation to the growth performance observed in the present study. The LW and ADG also increased linearly and/or quadratically till 10% of HI meal inclusion, and decreased (often dramatically) for the inclusion of 15% of insects. The overall FCR also increased linearly with increasing levels of HI meal, reaching its maximum for the inclusion of 15% of insects. Furthermore, the alterations of the gut morphology can also explain the observed growth performance. In fact, considering that the rapid growth of chickens depends directly on the morphological and functional integrity of the digestive tract [25], the relationship between the negative gut morphometric indices and the deterioration of the growth performance seems logical, as already suggested by Biasato et al. [14]. Dietary HI larva meal inclusion did not affect the development or the severity of the histopathological alterations in the broilers of the present trial, thus suggesting no negative influence on animal health. This is in agreement with Biasato et al. [11, 13, 14], who observed no differences between free-range and broiler chickens fed diets which included TM larva meal. As already suggested by the same authors, the different degrees of lymphoid system activation identified in the birds of the current study could be related to some stressful situations that frequently occur in modern poultry rearing operations [56].

## Conclusions

The current study provides novel useful information on the use of black soldier fly as a supplemental source of protein in broiler diets at different levels. The increasing levels of dietary HI meal inclusion in broiler chicken diets may improve the LW and DFI of chickens during the starter period. Nevertheless, the results also indicate that it could negatively affect the FCR during the growing and finishing periods, as well as the intestinal morphology, especially for high levels of inclusion (i.e. 15%, thus making lower levels more suitable). However, no significant effects on the haematochemical parameters or histological features were observed. Further research is needed to confirm the results of this study. Moreover, new information acquired by this work could help the EC policy makers to give the green light on the use of insect meals in poultry feeds.

## Abbreviations

AA: Amino acid
ADG: Average daily gain
AMEn: Apparent metabolizable energy
AST: Aspartate amino transferease
Cd: Crypt depth
CF: Crude fiber
CP: Crude protein
DFI: Daily feed intake
DM: Dry matter
DU: Duodenum
EC: European commission
EE: Ether extract
FCR: Feed conversion ratio
GPx: Glutathione peroxidase
H/L: Heterophiles to lymphocytes ratio
Hb: Haemoglobin
HI: *Hermetia illucens*
JE: Jejunum
IL: Ileum
LW: Live weight
SEM: Standard error of the means
TAS: Total antioxidant status
Vh: Villus height
Vh/Cd: Villus height-to- crypt depth ratio
